# Supplementary material for: Changing bird communities of an agricultural landscape: declines in arboreal foragers, increases in large species
Source: R Soc Open Sci. 2020 Mar 11;7(3):200076. doi: 10.1098/rsos.200076 (PMC7137982; doi:10.1098/rsos.200076)

## Supplementary Material

**Table S1** List of bird species recorded during transect surveys.

| Common Name^a^ | Scientific Name | Conservation Status^b^ | Diet^c^ | Movement^d^ | Native Status^e^ | Body Size^f^ | Foraging Height^g^ |
| --- | --- | --- | --- | --- | --- | --- | --- |
| Australian Hobby | ***Falco longipennis*** | *LC* | Vertebrates | R | Nat.Tas | L | Aer |
| Southern Australian Hobby | *Falco longipennis longipennis* | *LC* |  |  |  |  |  |
| Australian Magpie | ***Gymnorhina tibicen*** | *LC* | Invertebrates | R | Nat.Tas | L | Ter |
| Tasmanian Australian Magpie | *Gymnorhina tibicen hypoleuca* | *LC* |  |  |  |  |  |
| Australian Shelduck | ***Tadorna tadornoides*** | *LC* | Invertebrates, Plants | N | Nat.Tas | VL | AqTer |
| Australian Wood Duck | ***Chenonetta jubata*** | *LC* | Invertebrates, Plants | N | Nat.Tas | L | AqTer |
| Beautiful Firetail | ***Stagonopleura bella*** | *LC* | Seeds | R | Nat.Tas | S | Ter |
| South-eastern Beautiful Firetail | *Stagonopleura bella bella* | *LC* |  |  |  |  |  |
| Black Currawong | ***Strepera fuliginosa*** | *LC* | Invertebrates, Vertebrates | N | End.Tas | L | Ter |
| Tasmanian Black Currawong | *Strepera fuliginosa fuliginosa* | *LC* |  |  |  |  |  |
| Black-faced Cuckoo-shrike | ***Coracina novaehollandiae*** | *LC* | Invertebrates | M | Nat.Tas | L | Arb |
| Tasmanian Black-faced Cuckoo-shrike | *Coracina novaehollandiae novaehollandiae* | *LC* |  |  |  |  |  |
| Black-headed Honeyeater | ***Melithreptus affinis*** | *LC* | Invertebrates | R | End.Tas | S | Arb |
| Blue-winged Parrot | ***Neophema chrysostoma*** | *LC* | Seeds | M | Nat.Tas | M | Ter |
| Brown Falcon | ***Falco berigora*** | *LC* | Vertebrates | R* | Nat.Tas | L | Aer |
| Australian Brown Falcon | *Falco berigora berigora* | *LC* |  |  |  |  |  |
| Brown Goshawk | ***Accipiter fasciatus*** | *LC* | Vertebrates | R* | Nat.Tas | L | Aer |
| Southern Brown Goshawk | *Accipiter fasciatus fasciatus* | *LC* |  |  |  |  |  |
| Brown Quail | ***Synoicus ypsilophorus*** | *LC* | Invertebrates, Seeds | R | Nat.Tas | L | Ter |
| Tasmanian Brown Quail | *Synoicus ypsilophorus ypsilophorus* | *LC* |  |  |  |  |  |
| Brown Thornbill | ***Acanthiza pusilla*** | *LC* | Invertebrates | R | Nat.Tas | S | Arb |
| Tasmanian Brown Thornbill | *Acanthiza pusilla diemenensis* | *LC* |  |  |  |  |  |
| Brush Bronzewing | ***Phaps elegans*** | *LC* | Seeds | R | Nat.Tas | L | Ter |
| Eastern Brush Bronzewing | *Phaps elegans elegans* | *LC* |  |  |  |  |  |
| Chestnut Teal | ***Anas castanea*** | *LC* | Invertebrates, Plants | N | Nat.Tas | L | Aqu |
| Collared Sparrowhawk | ***Accipiter cirrocephalus*** | *LC* | Vertebrates | N | Nat.Tas | L | Aer |
| Australian Collared Sparrowhawk | *Accipiter cirrocephalus cirrhocephalis* | *LC* |  |  |  |  |  |
| Common Blackbird | ***Turdus merula*** | *I* | Invertebrates | R | Exo.Aus | M | Ter |
| Common Bronzewing | ***Phaps chalcoptera*** | *LC* | Seeds | R | Nat.Tas | L | Ter |
| Common Pheasant | ***Phasianus colchicus*** | *I* | Invertebrates, Plants | R | Exo.Aus | VL | Ter |
| Common Starling | ***Sturnus vulgaris*** | *I* | Invertebrates | R | Exo.Aus | M | Ter |
| Crescent Honeyeater | ***Phylidonyris pyrrhopterus*** | *LC* | Nectar | N | Nat.Tas | S | Arb |
| Eastern Crescent Honeyeater | *Phylidonyris pyrrhopterus pyrrhopterus* | *LC* |  |  |  |  |  |
| Dusky Robin | ***Melanodryas vittata*** | *LC* | Invertebrates | R | End.Tas | M | Ter |
| Tasmanian Dusky Robin | *Melanodryas vittata vittata* | *LC* |  |  |  |  |  |
| Dusky Woodswallow | ***Artamus cyanopterus*** | *LC* | Invertebrates | M | Nat.Tas | M | Aer |
| Eastern Dusky Woodswallow | *Artamus cyanopterus cyanopterus* | *LC* |  |  |  |  |  |
| Eastern Rosella | ***Platycercus eximius*** | *LC* | Seeds | R | Nat.Tas | M | Ter |
| Tasmanian Eastern Rosella | *Platycercus eximius diemenensis* | *LC* |  |  |  |  |  |
| Eastern Spinebill | ***Acanthorhynchus tenuirostris*** | *LC* | Nectar | N | Nat.Tas | S | Arb |
| Tasmanian Eastern Spinebill | *Acanthorhynchus tenuirostris dubius* | *LC* |  |  |  |  |  |
| European Goldfinch | ***Carduelis carduelis*** | *I* | Seeds | R | Exo.Aus | S | Ter |
| Fan-tailed Cuckoo | ***Cacomantis flabelliformis*** | *LC* | Invertebrates | M | Nat.Tas | M | Arb |
| Australian Fan-tailed Cuckoo | *Cacomantis flabelliformis flabelliformis* | *LC* |  |  |  |  |  |
| Flame Robin | ***Petroica phoenicea*** | *NT* | Invertebrates | M | Nat.Tas | S | Ter |
| Forest Raven | ***Corvus tasmanicus*** | *LC* | Invertebrates, Vertebrates | R | Nat.Tas | L | ArTer |
| Southern Forest Raven | *Corvus tasmanicus tasmanicus* | *LC* |  |  |  |  |  |
| Galah | ***Eolophus roseicapilla*** | *LC* | Seeds | R | Nat.Tas* | L | Ter |
| Eastern Galah | *Eolophus roseicapilla albiceps* | *LC* |  |  |  |  |  |
| Golden Whistler | ***Pachycephala pectoralis*** | *LC* | Invertebrates | R | Nat.Tas | M | Arb |
| Tasmanian Golden Whistler | *Pachycephala pectoralis glaucura* | *LC* |  |  |  |  |  |
| Common Greenfinch | ***Chloris chloris*** | *I* | Seeds | R | Exo.Aus | M | Ter |
| Green Rosella | ***Platycercus caledonicus*** | *LC* | Seeds | R | End.Tas | L | Arb |
| Tasmanian Green Rosella | *Platycercus caledonicus caledonicus* | *LC* |  |  |  |  |  |
| Grey Butcherbird | ***Cracticus torquatus*** | *LC* | Invertebrates, Vertebrates | R | Nat.Tas | M | ArTer |
| Tasmanian Grey Butcherbird | *Cracticus torquatus cinereus* | *LC* |  |  |  |  |  |
| Grey Currawong | ***Strepera versicolor*** | *LC* | Invertebrates, Vertebrates | R | Nat.Tas | L | ArTer |
| Tasmanian Grey Currawong | *Strepera versicolor arguta* | *LC* |  |  |  |  |  |
| Grey Fantail | ***Rhipidura fuliginosa*** | *LC* | Invertebrates | N | Nat.Tas | S | Arb |
| Tasmanian Grey Fantail | *Rhipidura fuliginosa albiscapa* | *LC* |  |  |  |  |  |
| Grey Goshawk | ***Accipiter novaehollandiae*** | *LC* | Vertebrates | R | Nat.Tas | L | Aer |
| Grey Shrike-thrush | ***Colluricincla harmonica*** | *LC* | Invertebrates, Vertebrates | R | Nat.Tas | M | ArTer |
| Tasmanian Grey Shrike-thrush | *Colluricincla harmonica strigata* | *LC* |  |  |  |  |  |
| House Sparrow | ***Passer domesticus*** | *I* | Invertebrates, Seeds | R | Exo.Aus | M | Ter |
| Laughing Kookaburra | ***Dacelo novaeguineae*** | *LC* | Invertebrates, Vertebrates | R | Exo.Tas | L | Arb |
| Southern Laughing Kookaburra | *Dacelo novaeguineae novaeguineae* | *LC* |  |  |  |  |  |
| Little Wattlebird | ***Anthochaera chrysoptera*** | *LC* | Nectar | N | Nat.Tas | M | Arb |
| Tasmanian Little Wattlebird | *Anthochaera chrysoptera tasmanica* | *LC* |  |  |  |  |  |
| Little Corella | ***Cacatua sanguinea*** | *LC* | Seeds | R | Exo.Tas | L | Ter |
| Eastern Little Corella | *Cacatua sanguinea gymnopis* | *LC* |  |  |  |  |  |
| Long-billed Corella | ***Cacatua tenuirostris*** | *LC* | Seeds | R | Exo.Tas | L | Ter |
| Musk Lorikeet | ***Glossopsitta concinna*** | *LC* | Nectar | N | Nat.Tas | M | Arb |
| Tasmanian Musk Lorikeet | *Glossopsitta concinna didimus* | *LC* |  |  |  |  |  |
| Nankeen Kestrel | ***Falco cenchroides*** | *LC* | Vertebrates | M | Nat.Tas | L | Aer |
| Australasian Nankeen Kestrel | *Falco cenchroides cenchroides* | *LC* |  |  |  |  |  |
| New Holland Honeyeater | ***Phylidonyris novaehollandiae*** | *LC* | Nectar | R | Nat.Tas | S | Arb |
| Tasmanian New Holland Honeyeater | *Phylidonyris novaehollandiae canescens* | *LC* |  |  |  |  |  |
| Noisy Miner | ***Manorina melanocephala*** | *LC* | Invertebrates | R | Nat.Tas | M | Arb |
| Tasmanian Noisy Miner | *Manorina melanocephala leachi* | *LC* |  |  |  |  |  |
| Olive Whistler | ***Pachycephala olivacea*** | *LC* | Invertebrates | R | Nat.Tas | M | Arb |
| Tasmanian Olive Whistler | *Pachycephala olivacea apatetes* | *LC* |  |  |  |  |  |
| Pacific Black Duck | ***Anas superciliosa*** | *LC* | Invertebrates, Plants | N | Nat.Tas | VL | Aqu |
| Pallid Cuckoo | ***Heteroscenes pallidus*** | *LC* | Invertebrates | M | Nat.Tas | M | Arb |
| Pink Robin | ***Petroica rodinogaster*** | *LC* | Invertebrates | R | Nat.Tas | S | Ter |
| Tasmanian Pink Robin | *Petroica rodinogaster rodinogaster* | *LC* |  |  |  |  |  |
| Satin Flycatcher | ***Myiagra cyanoleuca*** | *LC* | Invertebrates | M | Nat.Tas | S | Arb |
| Scarlet Robin | ***Petroica multicolor*** | *LC* | Invertebrates | R | Nat.Tas | S | Ter |
| Tasmanian Scarlet Robin | *Petroica multicolor leggii* | *LC* |  |  |  |  |  |
| Shining Bronze-Cuckoo | ***Chalcites lucidus*** | *LC* | Invertebrates | M | Nat.Tas | M | Arb |
| Australian Shining Bronze-Cuckoo | *Chalcites lucidus plagosus* | *LC* |  |  |  |  |  |
| Silvereye | ***Zosterops lateralis*** | *LC* | Invertebrates | M | Nat.Tas | S | Arb |
| Tasmanian Silvereye | *Zosterops lateralis lateralis* | *LC* |  |  |  |  |  |
| Spotted Pardalote | ***Pardalotus punctatus*** | *LC* | Invertebrates | R | Nat.Tas | S | Arb |
| Coastal Spotted Pardalote | *Pardalotus punctatus punctatus* | *LC* |  |  |  |  |  |
| Striated Pardalote | ***Pardalotus striatus*** | *LC* | Invertebrates | M | Nat.Tas | S | Arb |
| Tasmanian Striated Pardalote | *Pardalotus striatus striatus* | *LC* |  |  |  |  |  |
| Strong-billed Honeyeater | ***Melithreptus validirostris*** | *LC* | Invertebrates | R | End.Tas | M | Arb |
| Sulphur-crested Cockatoo | ***Cacatua galerita*** | *LC* | Seeds | R | Nat.Tas | L | Ter |
| Eastern Sulphur-crested Cockatoo | *Cacatua galerita galerita* | *LC* |  |  |  |  |  |
| Superb Fairy-wren | ***Malurus cyaneus*** | *LC* | Invertebrates | R | Nat.Tas | S | Ter |
| Tasmanian Superb Fairy-wren | *Malurus cyaneus cyaneus* | *LC* |  |  |  |  |  |
| Swamp Harrier | ***Circus approximans*** | *LC* | Vertebrates | M | Nat.Tas | L | Aer |
| Tasmanian Scrubwren | ***Sericornis humilis*** | *LC* | Invertebrates | R | End.Tas | S | Ter |
| Southern Tasmanian Scrubwren | *Sericornis humilis humilis* | *LC* |  |  |  |  |  |
| Tree Martin | ***Petrochelidon nigricans*** | *LC* | Invertebrates | M | Nat.Tas | S | Aer |
| Tasmanian Tree Martin | *Petrochelidon nigricans nigricans* | *LC* |  |  |  |  |  |
| Wedge-tailed Eagle | ***Aquila audax*** | *LC* | Vertebrates | R | Nat.Tas | VL | Aer |
| Tasmanian Wedge-tailed Eagle | *Aquila audax fleayi* | *V* |  |  |  |  |  |
| Welcome Swallow | ***Hirundo neoxena*** | *LC* | Invertebrates | M | Nat.Tas | S | Aer |
| Eastern Welcome Swallow | *Hirundo neoxena neoxena* | *LC* |  |  |  |  |  |
| White-faced Heron | ***Egretta novaehollandiae*** | *LC* | Vertebrates | N | Nat.Tas | L | Aqu |
| White-fronted Chat | ***Epthianura albifrons*** | *LC* | Invertebrates | N | Nat.Tas | S | Ter |
| Yellow Wattlebird | ***Anthochaera paradoxa*** | *LC* | Nectar | N* | End.Tas | L | Arb |
| Tasmanian Yellow Wattlebird | *Anthochaera paradoxa paradoxa* | *LC* |  |  |  |  |  |
| Yellow-rumped Thornbill | ***Acanthiza chrysorrhoa*** | *LC* | Invertebrates | R* | Nat.Tas | S | Ter |
| Tasmanian Yellow-rumped Thornbill | *Acanthiza chrysorrhoa leachi* | *LC* |  |  |  |  |  |
| Yellow-tailed Black-Cockatoo | ***Zanda funereus*** | *LC* | Seeds | N | Nat.Tas | L | Arb |
| Tasmanian Yellow-tailed Black-Cockatoo | *Zanda funereus xanthanota* | *LC* |  |  |  |  |  |
| Yellow-throated Honeyeater | ***Nesoptilotis flavicollis*** | *LC* | Invertebrates | R | End.Tas | M | Arb |

^a^ Species level names are in bold and the relevant subspecies is listed below. All names are derived from the Working List of Australian Birds Version 2.1.

^b^ Australian conservation status is derived from the Working List of Australian Birds. LC = least concern, V = vulnerable, NT = near threatened, EN = endangered, CE = critically endangered, I = introduced

^c^ Diet information is derived from the Handbook of Australian, New Zealand and Antarctic Birds.

^d^ Movement information is derived from the Handbook of Australian, New Zealand and Antarctic Birds. Asterisk denotes that knowledge of migration is unknown, unclear or varies by geographic region. M = Tasmanian migrant, R = resident or sedentary, N = nomadic (including some altitudinal migrants).

^e^ Nat.Tas = native to Tasmania, End.Tas = endemic Tasmanian species, Exo.Tas = exotic to Tasmania from the Australian mainland, Exo.Aus = exotic to Australia including Tasmania. Conflicting information exists on whether galahs are native to Tasmania or not.

^f^ Body size was classified as described in Table 2. S = small, M = medium, L = large, VL = very large.

^g^ Arb = arboreal, Aer = aerial forager, Aqu = aquatic forager, Ter = terrestrial / ground forager, ArTer = species that forage both arboreally and on the ground, AqTer = species that forage both on water and on the ground.

**Table S2.** List of species recorded only during 2 ha / 20 minute surveys of woodland or planting sites.

| **Common Name^a^** | **Scientific Name** | **Conservation Status^b^** | **Diet^c^** | **Movement^d^** | **Native Status^e^** | **Body Size^f^** | **Foraging Height^g^** |
| --- | --- | --- | --- | --- | --- | --- | --- |
| **Tawny Frogmouth** | ***Podargus strigoides*** | *LC* | Invertebrates, Vertebrates | R | Nat.Tas | L | Arb |
| Eastern Tawny Frogmouth | *Podargus strigoides strigoides* | *LC* |  |  |  |  |  |
| **Silver Gull** | ***Chroicocephalus novaehollandiae*** | *LC* | Invertebrates, Vertebrates | N | Nat.Tas | L | Aqu |
| Australian Silver Gull | *Chroicocephalus novaehollandiae novaehollandiae* | *LC* |  |  |  |  |  |
| **White-bellied Sea-Eagle** | ***Haliaeetus leucogaster*** | *LC* | Vertebrates | R | Nat.Tas | VL | Aer |
| **Tasmanian Native-hen** | ***Tribonyx mortierii*** | *LC* | Plants | R | End.Tas | VL | Ter |
| **Peregrine Falcon** | ***Falco peregrinus*** | *LC* | Vertebrates | R | Nat.Tas | L | Aer |
| Australian Peregrine Falcon | *Falco peregrinus macropus* | *LC* |  |  |  |  |  |
| **Masked Lapwing** | ***Vanellus miles*** | *LC* | Invertebrates | R | Nat.Tas | L | Ter |
| Southern Masked Lapwing | *Vanellus miles novaehollandiae* | *LC* |  |  |  |  |  |
| **Grey Teal** | ***Anas gracilis*** | *LC* | Invertebrates, Plants | N | Nat.Tas | L | Aqu |
| **Great Cormorant** | ***Phalacrocorax carbo*** | *LC* | Vertebrates | N | Nat.Tas | VL | Aqu |
| Australian Great Cormorant | *Phalacrocorax carbo carboides* | *LC* |  |  |  |  |  |

**Table S3.** List of species recorded only during 2 ha / 20 minute surveys of native grasslands or pasture sites.

| **Common Name^a^** | **Scientific Name** | **Conservation Status^b^** | **Diet^c^** | **Movement^d^** | **Native Status^e^** | **Body Size^f^** | **Foraging Height^g^** |
| --- | --- | --- | --- | --- | --- | --- | --- |
| **Striated Fieldwren** | ***Calamanthus fuliginosus*** | *LC* | Invertebrates | R | Nat.Tas | S | Ter |
| Eastern Tasmanian Striated Fieldwren | *Calamanthus fuliginosus fuliginosus* | *LC* |  |  |  |  |  |
| **Eurasian Skylark** | ***Alauda arvensis*** | *I* | Invertebrates, Seeds | R | Exo.Aus | M | Ter |
| **Banded Lapwing** | ***Vanellus tricolor*** | *LC* | Invertebrates, Plants | N | Nat.Tas | L | Ter |
| **Australasian Pipit** | ***Anthus novaeseelandiae*** | *LC* | Invertebrates, Seeds | N | Nat.Tas | M | Ter |
| Tasmanian Australian Pipit | *Anthus novaeseelandiae bistriatus* | *LC* |  |  |  |  |  |

**Table S4.** List of species heard offsite during surveys or that were recorded incidentally.

| **Common Name^a^** | **Scientific Name** | **Conservation Status^b^** | **Diet^c^** | **Movement^d^** | **Native Status^e^** | **Body Size^f^** | **Foraging Height^g^** |
| --- | --- | --- | --- | --- | --- | --- | --- |
| **Turkey** | ***Meleagris*** | *I* | Plants | R | Exo.Aus | VL | Ter |
| **Swift Parrot** | ***Lathamus discolor*** | *CE* | Nectar | M | Nat.Tas | M | Arb |
| **Spotted Dove** | ***Streptopelia chinensis*** | *I* | Seeds | R | Exo.Aus | L | Ter |
| **Red Junglefowl** | ***Gallus gallus*** | *I* | Invertebrates, Plants | R | Exo.Aus | L | Ter |
| **Indian Peafowl** | ***Pavo cristatus*** | *I* | Invertebrates, Plants | R | Exo.Aus | VL | Ter |
| **Helmeted Guineafowl** | ***Numida meleagris*** | *I* | Invertebrates, Seeds | R | Exo.Aus | VL | Ter |
| **Horsfield's Bronze-Cuckoo** | ***Chalcites basalis*** | *LC* | Invertebrates | M | Nat.Tas | S | Ter |
| **Black Swan** | ***Cygnus atratus*** | *LC* | Plants | R | Nat.Tas | VL | Aqu |
| **Masked Owl** | ***Tyto novaehollandiae*** | *LC* | Vertebrates | R | Nat.Tas | VL | Aer |
| Tasmanian Masked Owl | *Tyto novaehollandiae castanops* | *EN* |  |  |  |  |  |
| **Tasmanian Boobook** | ***Ninox leucopsis*** | *LC* | Vertebrates | M* | Nat.Tas | L | Aer |
| **Tasmanian Thornbill** | ***Acanthiza ewingii*** | *LC* | Invertebrates | R | End.Tas | S | Arb |
| Southern Tasmanian Thornbill | *Acanthiza ewingii ewingii* | *LC* |  |  |  |  |  |

**Figure S1.** Change in native species richness and bird density (birds hectare^-1^) at historical survey sites between the 1997 and 2017 survey periods.


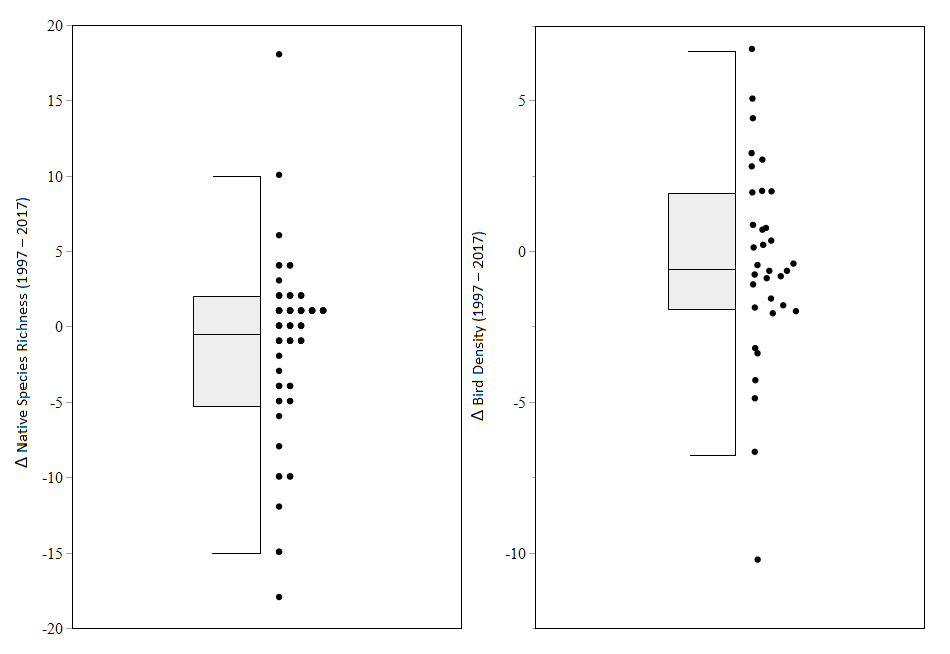


**Table S5.** Models with weight < 0.10 of the relationship between change in species richness at historical survey sites and changes in noisy miner density, woody vegetation cover, patch size, change in patch size and the number of centre pivot irrigators within 1 km.

| Δ Native Species Richness | | | | | | | |  |
| --- | --- | --- | --- | --- | --- | --- | --- | --- |
| AIC*c* | Δ AIC*c* | *W* | Δ Noisy Miner Density | Δ Woody  Vegetation Cover | Patch Size | Δ Patch Size | Pivot Irrigators | |
| 213.18 | 2.82 | 0.09 | -5.415 ± 1.393 | 0.387 ± 0.184 | 0.004 ± 0.002 | - | 0.261 ± 0.648 | |
| 215.08 | 4.72 | 0.04 | -5.819 ± 1.440 | 0.435 ± 0.189 | 0.004 ± 0.002 | 0.251 ± 0.237 | 0.289 ± 0.647 | |
| 221.24 | 10.88 | 0.00 | - | 0.537 ± 0.217 | - | - | - | |
| 225.74 | 15.38 | 0.00 | - | - | 0.003 ± 0003 | - | - | |
| 230.53 | 20.17 | 0.00 | - | - | 0.003 ± 0.003 | -0.201 ± 0.293 | 0.203 ± 0.293 | |

**Figure S2.** Change in native bird species richness at historical survey sites (*n* = 33) plotted against their initial richness in the 1997 survey period. Colour of data points indicates the change in noisy miner density (miners hectare^-1^) at each site. The dashed line indicates a zero net change in species richness.


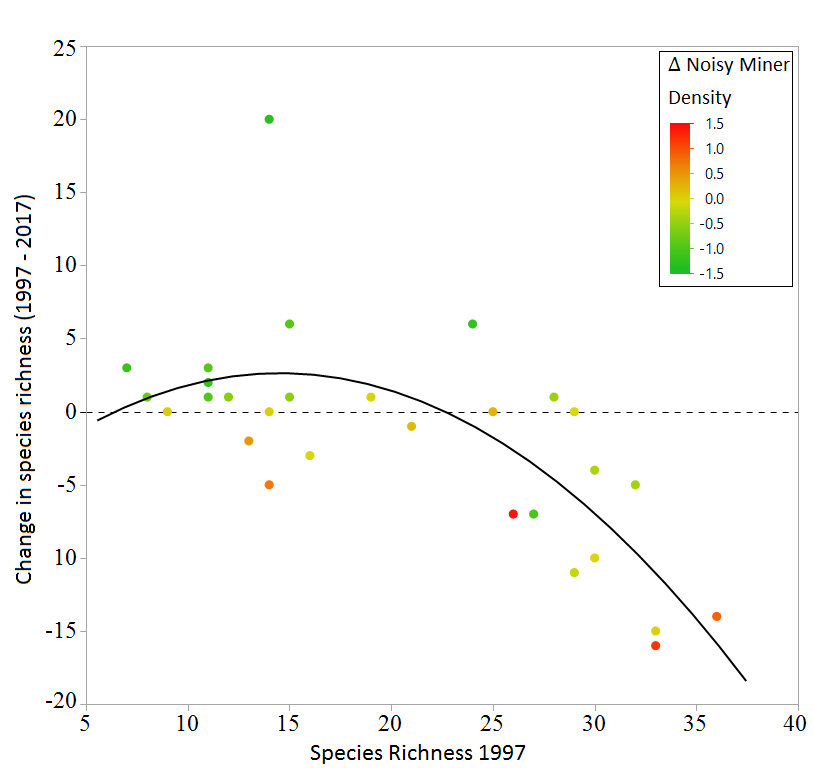

Supplement: Species list & additional models of change in species richness [file rsos200076supp1.docx]
